# Differential regulation of c-Met signaling pathways for synovial cell function

**DOI:** 10.1186/2193-1801-3-554

**Published:** 2014-09-23

**Authors:** Seiji Shibasaki, Sachi Tsunemi, Sachie Kitano, Masahiro Sekiguchi, Hajime Sano, Tsuyoshi Iwasaki

**Affiliations:** General Education Center, Hyogo University of Health Sciences, 1-3-6 Minatojima, Chuo-ku, Kobe, 650-8530 Japan; Division of Rheumatology, Department of Internal Medicine, Hyogo College of Medicine, 1-1 Mukogawa-cho, Nishinomiya, Hyogo, 663-8501 Japan; Division of Pharmacotherapy, Department of Pharmacy, School of Pharmacy, Hyogo University of Health Sciences, 1-3-6 Minatojima, Chuo-ku, Kobe, 650-8530 Japan

**Keywords:** c-Met, Hepatocyte growth factor, Rheumatoid arthritis, Synovial cell

## Abstract

We previously demonstrated that blocking the hepatocyte growth factor (HGF) receptor, c-Met, using a HGF antagonist, NK4, inhibited arthritis in a rheumatoid arthritis (RA) model mice. In the present study, we investigated the role of c-Met signaling in synovial cell function. We demonstrated that synovial tissues from RA patients and MH7A cells, a human RA synovial cell line, expressed HGF and c-Met. HGF and c-Met expression in RA synovium was increased compared to osteoarthritis synovium suggesting increased c-Met signaling in RA synovial cells. The c-Met inhibitor, SU11274, inhibited ERK1/2 and AKT phosphorylation in HGF-stimulated MH7A cells. MEK and PI3K inhibitors suppressed production of matrix metalloproteinase-3 (MMP-3), vascular endothelial growth factor (VEGF) and prostaglandin E2 (PGE2) by MH7A cells, suggesting that c-Met-MEK-ERK and c-Met-PI3K-AKT pathways are involved positively regulating MH7A cell function. Although SU11274 suppressed MMP-3 and VEGF production it enhanced PGE2 production by MH7A cells suggesting that negative regulation by c-Met signaling, independent of the MEK-ERK and PI3K-AKT pathways, is involved in PGE2 production. Blocking c-Met signaling may be therapeutically useful to inhibit angiogenesis and cartilage and bone destruction by inhibiting VEGF and MMP-3 production, while enhancing PGE2 production in synovial cells in RA.

## Introduction

Rheumatoid arthritis (RA) is characterized by an aggressive synovial expansion due to angiogenesis and subsequent destruction of the underlying cartilage and bone (Szekanecz and Koch 2008, Scot et al. 2010, Szekanecz et al. 2010). Synovial cells produce inflammatory mediators, proteases, and angiogenic growth factors such as prostaglandin E2 (PGE2), matrix metalloproteinases (MMPs), and vascular endothelial growth factor (VEGF) (Pap et al. 2000).

MMPs are responsible for the destruction of cartilage and bone in RA joints. In particular, MMP-1 and MMP-3 are critical proteases in tissue degradation in RA (Walakovits et al. 1992, Green et al. 2003). PGE2 plays a major role in angiogenesis through the expression of VEGF in rheumatoid synovium (Ben-Av et al. 1995). Moreover, PGE2 has been shown to trigger bone resorption by osteoclasts (Mino et al. 1998) suggesting that the inflammation of RA is closely related to the production of PGE2 by synovial cells (Sano et al. 1992). TNF-a is a well-known cytokine that stimulates the production of MMPs and PGE2 through activation of intracellular signaling pathways including mitogen-activated protein kinase (MAPK) and activator protein-1 (AP-1) (Han et al. 2001, Reunanen et al. 2002, Martel-Pelletier et al. 2003, Akaogi et al. 2006).

New blood vessel formation is critically important for synovial expansion in RA. Angiogenic growth factors, such as VEGF and hepatocyte growth factor (HGF), enhance angiogenesis and induce synovial cell proliferation (Nagashima et al. 2001, Szekanecz et al. 2009, 2010). The HGF receptor, c-Met, appears to induce synovial cell proliferation in RA. We previously demonstrated that blocking c-Met signaling using a HGF antagonist, NK4, inhibited arthritis in a RA model of SKG mice (Tsunemi et al. 2013).

SU11274 is a prototypic anti-c-Met small molecule and an effective inhibitor of HGF-dependent signaling. SU11274 abrogates HGF-induced phosphorylation of c-Met and its downstream signal (Sattler et al. 2003). In the present study, we examined the effect of blocking c-Met signaling using NK4 or SU11274 on synovial cell function using a human synovial cell line *in vitro*.

## Materials and methods

### Cell line and cell culture

MH7A synovial cells isolated from intra-articular soft tissues of the knee joints of RA patients were obtained from Riken Cell Bank (Saitama, Japan). MH7A is a cell line established by transfecting with the SV40 T antigen (Miyazawa et al. 1988). MH7A cells were cultured in RPMI 1640 (Sigma, St. Louis, MO) containing 10% heat-inactivated fetal bovine serum (FBS) (Whittaker, Walkersville, MD), 100 units/ml of penicillin and 100 μg/ml of streptomycin (Invitrogen, San Diego, CA) at 37°C in an atmosphere of 5% CO_2_ in air.

### Immunohistochemical analysis of HGF and c-Met expression in RA synovium

Synovial tissue specimens isolated from patients with RA and osteoarthritis (OA) at the time of arthroscopic biopsy or total joint replacement were stained as reported previously (Kitano et al. 2006). Briefly, synovial tissue specimens were preserved in 10% formalin, embedded in paraffin, and serially sectioned onto microscope slides at a thickness of 4 μm. Synovial tissues were stained with either anti-human c-Met antibody (1:200 dilution in PBS, rabbit IgG: Santa Cruz Biotechnology) or anti-human HGF antibody (1:200 dilution in PBS, rabbit IgG: Santa Cruz Biotechnology), washed, incubated with biotinylated goat anti-rabbit IgG (1:1000 dilution in PBS: Santa Cruz Biotechnology), washed, incubated with avidin-biotinylated horseradish peroxidase complex (ABC) and diaminobenzidine tetrahydrochloride ([DAB], Elite kit; Vector Laboratories, Inc., Burlingame, CA, USA), and counterstained with Mayer’s hematoxylin. All patients gave informed consent, and the Institutional Medical Ethics Committee approved the study protocol.

### Western blot analysis

Western blot analysis was performed as reported previously (Dong et al. 2001). Briefly, MH7A cells (1 × 10^7^ cells/well) were lysed in RIPA lysis buffer (Santa Cruz Biotechnology, CA), and protein content was determined using Bio-Rad protein assay reagent (Bio-Rad, Hercules, CA) with bovine serum albumin as a standard. Each sample (20 μg) was resolved on 1% polyacrylamide gels under denaturing conditions and then transferred to 0.45-μm nitrocellulose membranes. After blocking overnight at 4°C with 5% nonfat milk in Tris-buffered saline-0.01% Tween 20 (Santa Cruz Biotechnology), membranes were incubated with primary antibodies against either phospho-extracellular signal-regulated kinase (ERK) 1/2, phospho-AKT, or β-actin (1:1000 dilution in PBS; Santa Cruz Biotechnology) after 12 h stimulation in the presence of HGF (10 ng/ml) with or without the c-Met inhibitor, SU11274 (Tocris Bioscience, UK). After washing the membranes with Tris buffered saline–0.05% Tween 20 (washing buffer), HRP-conjugated goat anti-rabbit secondary antibody (1:1000 dilution in PBS; Santa Cruz Biotechnology) was added, followed by incubation for 45 min. After further washing, the color was developed with luminol reagent (Santa Cruz Biotechnology), and HRP activity of the blots was analyzed using an LAS1000 imager. Luminol staining was done according to manufacturer’s instructions (Santa Cruz).

### Measurement of PGE2, VEGF and MMP-3 production by MH7A cells

MH7A cells (2 × 10^4^ cells/well) were plated in flat-bottomed 24-well microplates and cultured with or without either NK4 or c-Met inhibitor, SU11274 (Tocris Bioscience) in the presence or absence of 100 ng/ml TNF-α (Genzyme Pharmaceuticals, Cambridge, MA, USA). MH7A cells (2 × 10^4^ cells/well) were also cultured with or without either the MEK inhibitor, PD98059 (Cell Signaling Technology, MA), or the PI3K inhibitor, LY294002 (Cell Signaling Technology), in the presence or absence of 100 ng/ml TNF-α. After 72 hours of incubation, PGE2, VEGF and MMP-3 production was determined by assaying supernatants with ELISAs using either anti-human PGE2, VEGF and MMP-3 monoclonal antibodies (Genzyme), respectively, according to the manufacturer's protocol.

### Statistical analysis

Results are expressed as the mean ± SEM. The significance of the difference between the experimental results and control values was determined by a Student’s *t*-test. *P* values less than 0.05 were considered significant.

## Results

### HGF and c-Met expression in the synovium of RA patients

**Figure 1:**
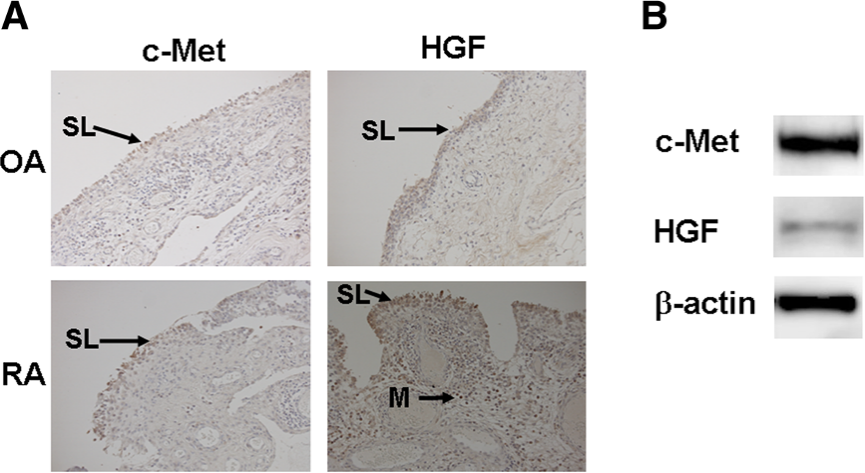
**c-Met and HGF expression in synovial cells. A**. Immunohistochemical analysis of joints. c-Met and HGF expression in synovial lining cells of RA patients was higher than in OA patients. HGF was expressed in infiltrating mononuclear cells (SL, synovial lining cell layer; M, mononuclear cells). Original magnification ×200. **B**. Western blot analysis of MH7A cells. c-Met and HGF were expressed in MH7A cells.

To examine whether c-Met signaling pathways are involved in the pathogenesis of RA, we examined the expression of HGF and c-Met in the synovium of OA and RA patients. Immunohistochemical analysis demonstrated that HGF and c-Met expression in synovial lining cells was higher in RA patients than in OA patients (Figure 1A). We also demonstrated that MH7A cells, a synovial cell line established from RA patients, expressed HGF and c-Met by Western blot analysis (Figure 1B). These results suggest that c-Met protein is increased in the synovial tissues of RA patients.

### SU11274 inhibits ERK1/2 and AKT phosphorylation by MH7A cells

**Figure 2:**
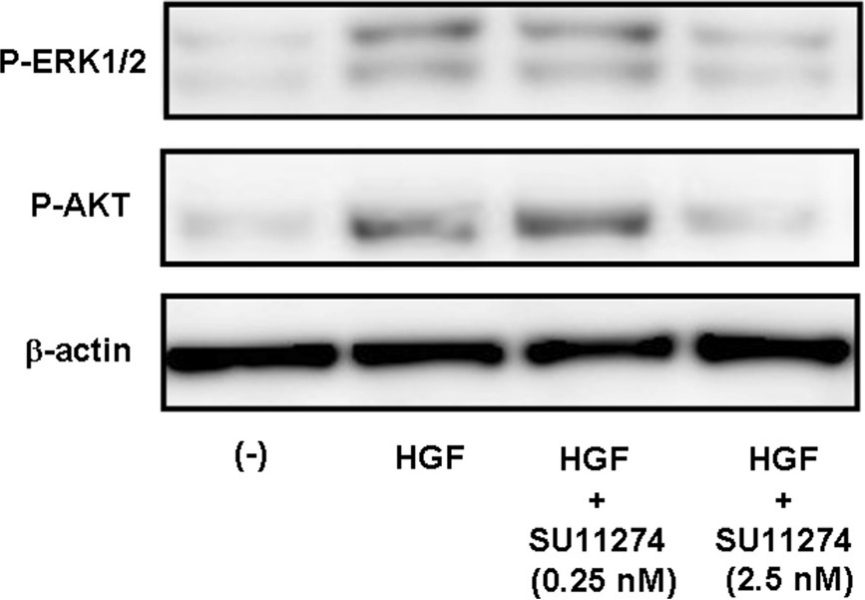
**Effects of the SU11274 inhibitor on ERK1/2 and AKT phosphorylation in MH7A cells.** MH7A cells were treated with HGF (10 ng/ml) with or without SU11274 (0.25 - 2.5 nM) and p-ERK1/2 and p-AKT expression was determined by Western blot analysis. We also determined β-actin expression as a control. Representative data of p-ERK1/2 and p-AKT expression by MH7A cells is shown.

MEK/ERK and PI3-AKT signaling pathways can be activated by a variety of growth factors such as insulin and HGF (Dong et al. 2001). To determine whether ERK or AKT was downstream of c-Met signaling, we examined the effect of the SU11274 inhibitor on ERK1/2 and AKT phosphorylation in response to HGF treatment. Stimulation of MH7A cells with HGF led to a significant increase in phosphorylation of ERK1/2 and AKT. Conversely, although treatment with the c-Met inhibitor (0.25 nM) did not affect treatment with the c-Met inhibitor (2.5 nM) reduced HGF-induced ERK1/2 and AKT phosphorylation, indicating that minimal dose of the c-Met inhibitor to block HGF-induced p-ERK1/2 and p-AKT expression is 0.25 nM (Figure 2). These results suggest that c-Met-MEK-ERK and c-Met-PI3-AKT signaling pathways are utilized in MH7A cells.

### Both MEK-ERK and PI3-AKT signaling pathways are involved in the production of MMP-3, VEGF and PGE2 by MH7A cells

**Figure 3:**
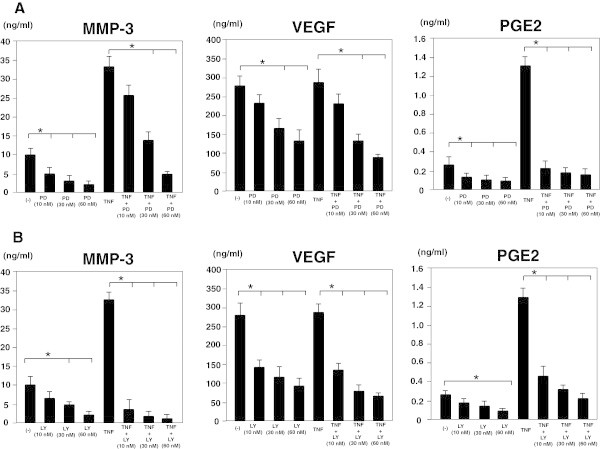
**Effects of MEK or PI3K inhibitors on the production of MMP-3, VEGF and PGE2 by MH7A cells. A**. MH7A cells (2 x 10^4^ cells/well) were cultured with the MEK inhibitor, PD98059 (10 - 60 nM), in the presence or absence of 100 ng/ml TNF-α. After 72 hours of incubation, MMP-3, VEGF and PGE2 production was determined by analysis of supernatants using ELISA. **B**. MH7A cells (2 x 10^4^ cells/well) were cultured with the PI3K inhibitor, LY294002 (10 - 60 nM), in the presence or absence of 100 ng/ml TNF-α. After 72 hours of incubation, MMP-3, VEGF and PGE2 production was determined by analysis of supernatants using ELISA. Data represent the mean ± SD of three independent experiments. *P < 0.01.

To confirm the involvement of MEK-ERK and PI3-AKT signaling pathways in the production of MMP-3, VEGF and PGE2, we examined whether pretreatment with either MEK or PI3K inhibitors affected MMP-3, VEGF and PGE2 production. Both the MEK inhibitor, PD98059 and the PI3K inhibitor, LY294002, inhibited MMP-3, VEGF and PGE2 production (Figure 3). These results suggest that both MEK-ERK and PI3-AKT signaling pathways are involved in MMP-3, VEGF and PGE2 production.

### Blocking c-Met signaling using NK4 or SU11274 inhibits MMP-3 and VEGF, but enhances PGE2 production by MH7A cells

**Figure 4:**
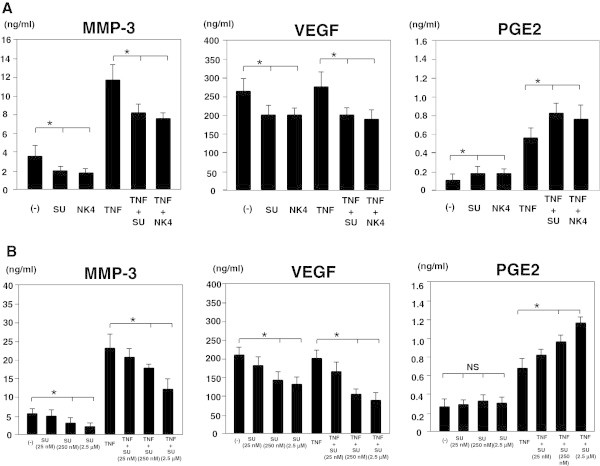
**Effects of blocking c-Met signaling on the production of MMP-3, VEGF, and PGE2 by MH7A cells. A**. MH7A cells (2 x 10^4^ cells/well) were cultured with or without either NK4 or SU11274 in the presence or absence of 100 ng/ml TNF-α. After 72 hours of incubation, MMP-3, VEGF and PGE2 production was determined by analysis of supernatants using ELISA. **B**. MH7A cells (2 x 10^4^ cells/well) were cultured with different dose of SU11274 in the presence or absence of 100 ng/ml TNF-α. After 72 hours of incubation, MMP-3, VEGF and PGE2 production was determined by analysis of supernatants using ELISA. Data represent the mean ± SD of three independent experiments. *P < 0.01. NS, not significant.

MH7A cells were treated with either NK4 or the c-Met inhibitor, SU11274, in the presence or absence of TNF-α for 72 hours and the production of MMP-3, VEGF and PGE2 was determined by ELISA. TNF-α significantly enhanced MMP-3 and PGE2, but not VEGF production by MH7A cells. Both NK4 and SU11274 inhibited MMP-3 and VEGF, but enhanced PGE2 production by MH7A cells (Figure 4A). We next examined the effect of different doses of SU11274 on MH7A cells. As shown in Figure 4B, SU11274 dose-dependently inhibited MMP-3 and VEGF, but enhanced PGE2 production by MH7A cells (Figure 4B). These results indicate that negative regulation by c-Met signaling independent of the MEK-ERK and PI3K-AKT pathway is also involved in PGE2 production.

## Discussion

We demonstrated that HGF and its receptor, c-Met, are expressed in synovial tissues and that both HGF and c-Met expression is increased in RA patients, but not OA patients. Synovial expansion is dependent on new blood vessel formation, which is important for the delivery of oxygen, nutrients, and inflammatory cells to the lesions of RA (Szekanecz and Koch 2008, Scot et al. 2010, Szekanecz et al. 2010). It has been reported that HGF and c-Met are expressed in synovial tissues in patients with RA (Nagashima et al. 2001). HGF in the synovial fluid of patients with RA is produced by synovial cells and is related to disease activity (Yukioka et al. 1994). Taken together, HGF may contribute to the development of RA by its potent angiogenic activities, which stimulate synovial cell proliferation.

We demonstrated that inhibition of c-Met signaling resulted in decreased VEGF and MMP-3 production by MH7A cells. VEGF is a potent angiogenic factor that is pivotal in RA pathogenesis. The role for HGF in VEGF production has been implicated in some cell types such as squamous cell carcinoma and synovial cells. It was demonstrated that c-Met signaling activates the PI3-AKT signaling pathway leading to up-regulation of VEGF expression (Dong et al. 2001, Lin et al. 2012). The present study demonstrated that c-Met signaling activates both the MEK-ERK and PI3-AKT signaling pathways leading to up-regulation of VEGF expression in the synovial cell line.

Esposito et al. (2009) have studied the anti-fibrogenic mechanisms of HGF in which increasing MMP expression promoted collagen degradation (Esposito et al. 2009). Addition of HGF to fibroblast cultures of scleroderma patients significantly decreased collagen production and increased MMP-1 expression and activity (Jinnin et al. 2005). Addition of HGF to keloid fibroblastic cells significantly increased the mRNA expression of MMP-1 and MMP-3 in a dose-dependent manner (Lee et al. 2011). This means that HGF may have a therapeutic effect on keloids that is similar to pathologic dermal fibrosis seen in scleroderma (Jinnin et al. 2005, Lee et al. 2011). In a previous study, we demonstrated that HGF gene transfection improved dermal sclerosis in mouse model of scleroderma (Iwasaki et al. 2006). The present results that the blocking c-Met signaling using the c-Met inhibitor (SU11274) inhibited MMP-3 production by MH7A cells provide support for the previous findings.

c-Met is a proto-oncogene that encodes a protein known as the HGF receptor (Liu et al. 1994, Zarnegar and Michalopoulos 1995). Upon HGF stimulation, c-Met induces c-Met kinase catalytic activity, which triggers transphosphorylation of tyrosines Tyr-1234 and Tyr-1235. These two tyrosines engage various signal transducers to initiate a whole spectrum of biological activities driven by c-Met. Two major signaling pathways, MEK/ERK and PI3/AKT, are activated by c-Met (Bottaro et al. 1991, Graziani et al. 1991, O'Brien et al. 2004, Mahtouk et al. 2010). We observed that both ERK1/2 and AKT phosphorylation was enhanced by HGF and was suppressed by the c-Met inhibitor. We also observed that both MEK and PI3K inhibitors suppressed VEGF and MMP-3 production. These results indicate that both c-Met-MEK/ERK and c-Met-PI3/AKT pathways are involved in the production of MMP-3 and VEGF by MH7A cells.

In contrast to the inhibitory effect of the c-Met inhibitor on MMP-3 and VEGF production, blocking c-Met signaling enhanced PGE2 production by MH7A cells. Previous work reported conflicting results that c-Met signaling can promote PGE2 production in colorectal cancer cells via COX-2 up-regulation and 15-PGDH down-regulation at the protein and messenger RNA level (Moore et al. 2009). They demonstrated that both ERK and AKT signals are required for COX-2 protein up-regulation and 15-PGDH down-regulation downstream of c-Met. Furthermore, SU11274 reduced COX-2 expression and increased 15-PGDH expression in high c-Met-expressing cells (Moore et al. 2009). We still do not know the mechanism by which the c-Met inhibitor enhances PGE2 production. Supporting our findings, Zhang et al., performed *in vitro* experiments on rabbit tendon cells and *in vivo* experiments in a mouse Achilles tendon injury model. They demonstrated that HGF treatment suppressed IL-1β-induced gene expression of COX-1, COX-2, and the production of PGE2 in tendon cell culture. Injection of HGF into wounded mouse Achilles tendons *in vivo* decreased PGE2 production in the tissues (Zhang et al. 2013).

HGF is known to function both as an anti-inflammatory agent and as an anti-fibrotic regulator (Gong 2008). Its anti-inflammatory action is primarily mediated by the disruption of signaling by the transcription factor, NF-kB, which is a critical regulator of inflammation. This was demonstrated both in an *in vivo* renal inflammation mouse model (Giannopoulou et al. 2008) and in human chondrocytes *in vitro* (Bendinelli et al. 2010). Such a c-Met-mediated negative signal for PGE2 production, independent of the c-Met-MEK-ERK and c-Met-PI3-AKT pathways, may be responsible for the anti-inflammatory effects of HGF on PGE2 production observed in this study. However, HGF has strong angiogenic activities which are crucial for synovial cell proliferation in RA. In this article, we suggested that blocking c-Met signaling inhibited angiogenesis and cartilage and bone destruction by the inhibition of VEGF and MMP-3 production by synovial cell line, respectively. We previously reported that blocking c-Met signaling using HGF antagonisit, NK4, inhibited arthritis in RA model of SKG mice (Tsunemi et al. 2013). Therefore, we think that although blocking c-Met signaling may enhances inflammatory responses, it is effective for the treatment of RA by its strong inhibition of angiogenesis and protease-mediated bone destruction.

In conclusion, this is the first report to demonstrate the differential regulation of c-Met signaling pathways for synovial cell function. Blocking c-Met signaling may be useful to inhibit angiogenesis and cartilage and bone destruction by inhibiting VEGF and MMP-3 production, respectively, while it enhances PGE2 production by synovial cells in RA.
